# Thermodynamics and Mechanics of Thermal Spraying of Steel EN 10060 Substrate with NiCrBSi Alloy after Milling

**DOI:** 10.3390/ma13235344

**Published:** 2020-11-25

**Authors:** Jan Valíček, Marta Harničárová, Jan Řehoř, Milena Kušnerová, Ludmila Kučerová, Miroslav Gombár, Jaroslava Fulemová, Jan Filipenský, Jan Hnátík

**Affiliations:** 1 Regional Technology Institute, Faculty of Mechanical Engineering, University of West Bohemia, Univerzitni 8, 306 14 Pilsen, Czech Republic; valicekj@rti.zcu.cz (J.V.); rehor4@kto.zcu.cz (J.Ř.); milena.kusnerova@gmail.com (M.K.); skal@rti.zcu.cz (L.K.); gombar@kto.zcu.cz (M.G.); fulemova@kto.zcu.cz (J.F.); jhnatik@kto.zcu.cz (J.H.); 2 PLASMAMETAL, Ltd., Tovární 917/1e, 643 00 Brno-Chrlice, Czech Republic; filipensky@plasmametal.cz

**Keywords:** coatings, HVOF, thermodynamics, mechanics, NiCrBSi alloy, thermal spraying

## Abstract

The objective of this paper is to present a new way of identifying and predicting the relationship between thermodynamic and physical-mechanical parameters in the formation of a layer after spraying on a substrate with NiCrBSi alloy and its subsequent processing by milling. The milling of the spherical surface of the EN 10060 material after spraying was performed on the DMU 40 eVolinear linear milling centre. The experimental part of the article is focused on investigating the influence of cutting parameters when machining a selected combination of materials (substrate-coating: EN 10060 steel-NiCrBSi alloy). The experiment is based on the results of direct measurements of three basic cutting parameters, namely: cutting speed *v_c_* (m∙min^−1^), feed per tooth *f_z_* (mm), and the depth of cut *a_p_* (mm). The new distribution functions of selected cutting parameters were derived. The analytical results of the thermodynamic calculations performed on nickel-based alloy can be used for accurate predictions of the technological parameters of milling a spherical substrate made of EN 10060 steel after HVOF spraying, and also for both sample preparation and the subsequent production of high-quality coatings.

## 1. Introduction

One of a number of thermal spray processes is high-velocity oxy-fuel coating spraying (HVOF). When spraying with a powder flame, the powdered material is melted by a flame of oxygen gas, it is accelerated toward the component by combustion gases, and is sprayed onto the surface of the part. Metal, oxide, ceramic, carbide, and plastic powder can be processed using spray guns specially designed for these materials. For metal alloys based on nickel, iron or cobalt, spray guns are chosen, which often take the form of hand burners, preferably using acetylene as the fuel gas due to its high flame temperature. The powder particles, which are partially melted by the flame, are deformed due to the impact with the surface of the component and are deposited on the surface of the substrate to form a spray coating with a lamellar structure. The main areas of application of thermal coatings are corrosion protection and wear protection. Powder flame spraying can be divided into cold and hot processes. In cold processes, the powders are applied only with a spray gun and the spray is not subjected to any subsequent heat treatment. In hot processes, metal powders of materials known as self-melting alloys based on Ni-Cr-B-Si are used. The sprayed layer is melted by subsequent heat treatment. The resulting thermal compaction makes it possible to obtain coatings which are practically free of pores. Therefore, depending on the application, two processes were created: simultaneous melting and subsequent melting.

Coatings produced by the HVOF process show many advantages. Because of the high particle velocity, a created HVOF coating has a low porosity (below 1%), a high bond strength (above 80 MPa) and a low surface roughness when comparing plasma or flame coatings. A detailed description of the HVOF process could be found in the fundamental monographs [1,2,3,4,5] as well as other papers [6,7].

Thermal spraying techniques are coating processes in which molten (or heated) materials are sprayed onto a surface. The “raw material” (coating precursor) is heated by electrical means (plasma or electric arc) or by chemical means (flame). Thermal spraying can provide thick coatings (approximately in the thickness range of 20 microns to several mm, depending on the process and raw material) over a large area at high application rates, compared to other coating processes (e.g., electroplating, physical and chemical vapour deposition). Materials available for thermal spraying include metals, alloys, ceramics, plastics and composites. They are fed in the form of powder or wire, heated to a molten or semi-liquid state and accelerated towards the substrate in the form of micron-sized particles. Combustion or electric arc are usually used as the energy source for thermal spraying. The resulting coatings are produced by the accumulation of numerous sprayed particles. The surface does not need to heat up significantly, which allows the coating of flammable substances. The quality of a coating is usually evaluated by measuring its porosity, oxide content, macro hardness and microhardness, bond strength, and surface roughness. In general, the quality of the coating increases with increasing particle velocity.

High entropy alloys are alloys that are formed by mixing equal or relatively large proportions, usually five or more elements. Prior to the synthesis of these substances, typical metal alloys contain one or two major components with minor amounts of other elements. For example, additional elements may be added to the iron to improve material properties. This forms an iron-based alloy, but usually in relatively low proportions, in particular, as is the case with the proportions of carbon and manganese in various steels. High entropy alloys are a new class of materials [8,9]. The term “highly entropic alloy” was coined because the increase in mixing entropy is significantly higher when there are a number of elements in the mixture, and their proportions are almost the same [10,11].

In applications where the wear resistance is required, the powders with carbide content and nickel base powders are used. Nickel-based alloys (self-fluxing alloy powders) play a crucial role in the field of corrosion protection. The common amount of Cr in alloys used for deposition of the protective coatings should be between 20 and 25 wt % [12,13]. During the deposition process, the temperature varies quickly, and this consequently affects the thermodynamics of the alloys. The effects of temperature conditions of substrate and particle on the process of coating formation have been studied in numerous works [14,15,16,17]. It has been proved that this point is very important in the formation of microstructure and thermo-mechanical properties of the coatings. The work Sobolev et al. [18] described a mathematical model used to predict the particle velocity, as well as temperature variations when using the HVOF spraying technology. Similar work was done by Planche et al. [19], who, moreover, compared the NiCrBSi powder prepared by plasma, flame, and the HVOF process. The remelting of NiCrBSi coatings achieved by the HVOF technology ensures a reduction of the porosity, and, consequently, it leads to the improvement of the mechanical and microstructure properties [20]. Machining of thermal-sprayed layers is a great challenge for many new companies, in particular for machining surfaces that are difficult to machine. Quality of machining of that kind of coated surfaces is dependent on the special coating material characteristics. The thermal-sprayed layer is considered as an inhomogeneous material. In order to avoid problems during machining of thermal-sprayed layers, a strong binding between the droplets binding must be guaranteed [21]. When the cutting force is bigger than the cohesive strength of the layer, the coating particles can be easily pulled out from the substrate surface [22]. Solutions dealing with the improvement of the machining performance of difficult-to-machine materials can be found in [23,24,25]. The selection of the right cutting tool geometry, its optimisation and analysis of properties of hard coating systems in machining these alloys is critical [26]. An effort to find the right combination of these parameters can lead to wear reduction and thus to the improvement of the machining. Barthelmä et al. [27] devoted their research to improve the milling of nickel-based alloys through the cutting tool optimisation (optimising the geometry of the cutting tools and coating) and process optimisation (simulating the cutting forces and temperatures). The adhesion and strength of a coating is a critical aspect that is related to machining performance.

However, up to now, there are no works that report on the thermodynamics and mechanics of thermal spraying of steel EN 10060 substrate with NiCrBSi alloy in connection with the milling technology. Therefore, a detailed study of the physical-mechanical and new thermodynamic functions is undoubtedly necessary and may bring new insight for further understanding of the mechanism of the machining process in connection with the melting processes given by the thermal spraying of the substrate.

The topic of the work reflects the requirements of practice, in connection with close cooperation with the company PlazmaMetal Ltd. (Brno, Czech Republic). This company performs thermal spraying in railway transport where the presented NiCrBSi alloy is used to protect the surfaces of railway components.

The NiCrBSi-based alloy is used for applications where good machinability is required and where its hardness is sufficient in terms of wear resistance. After remelting, the coating is gas-tight with diffusion bonding to the substrate. NiCrBSi alloy substrate material has good self-fluxing nature and wettability. Another advantage of using NiCrBSi alloys is their ability to enhance body/matrix properties, coating/substrate interface bonding properties, and also an advantageous price.

The main motivation of our work is that the generated surfaces need to be additionally modified to the required roughness after spraying. Therefore, the article places particular emphasis on monitoring the combination of technological parameters of machining in relation to the parameters of the generated coating.

The main hypothesis of our work is the expression of the entropy of the spray after the application of HVOF because it is due to melting that a modified structure characterised by high entropy is created.

The work is especially important as innovative and optimising because the research of this type through enthalpy and entropy allows describing the mechanism of surface layer formation and subsequent technological treatment by machining in an analytical way. With knowledge of this mechanism, we are able to arrange the inhomogeneous-highly entropic environment in such a way that it becomes reasonably homogeneous, at the cost of supplying energy-enthalpy dimensioned by the concerned models. Only a quasi-homogeneous layer meets the expectation that it will function as a good protective one. Only the setting of optimal technological parameters can help to implement the main, already formulated goal.

The genesis of the research is based on previous publications on cobalt and nickel alloys (Stellite 1,2, Stellite 6 as the Co-Cr-W), which were finally machined by turning [28,29,30]. In this research, we dealt with the interaction of individual parameters.

Attention should be focused on a comprehensive approach to the description of interactions between partial parameters, namely thermodynamic, mechanical and technological, because they are strongly interconnected and conditioned.

However, up to now, there have been no works reporting on the thermodynamics and mechanics of thermal spraying of steel EN 10060 substrate with NiCrBSi alloy in connection with the milling technology. Therefore, a detailed study of the physical-mechanical and new thermodynamic functions is undoubtedly necessary and may bring new insight for further understanding of the mechanism of the machining process in connection with the melting processes given by the thermal spraying of the substrate.

## 2. Materials and Methods

The experimental part of the article is focused on previous theoretical and experimental knowledge about the influence of specifically defined combinations of cutting parameters in the machining of spherical surfaces of selected materials for coating. Currently, the highly entropic spraying material NiCrBSi alloy is used, and steel EN 100 60 (34CrNiMo6) is used as substrate. In the presented experiments, a total of 15 cuts were milled on the coating sprayed using NiCrBSi alloy. A method of calculating the main mechanical parameters in discrete dependences on the choice of cutting parameters was proposed, based on three different combinations of cutting parameters, namely speed of cut *v_c_* (m∙min^−1^), feed per tooth *f* (mm), and depth of cut *a_p_* (mm). These machining parameters are considered technologically fundamental because they directly create the machined volume of material, structure and texture of the machined surface, its instantaneous stress-strain state, and, in the case of thermal spraying, a specific state of thermodynamic quantities at the substrate-spray contact. The mutual layering of spray layers, their thermodynamic and mechanical transitions, are investigated in more detail analytically. The theoretical way of solving mechanical and thermodynamic problems of the spraying process, which is derived on the basis of experimental results, is also presented and verified on the manufactured workpiece. The differentiated interfaces of the individual layers, and thus their thickness, are clearly visible on the edges of the workpiece under a microscope or even only under a magnifying glass. These data can be well used to verify the theoretically calculated material and dimensional parameters of individual spray layers.

**Figure 1:**
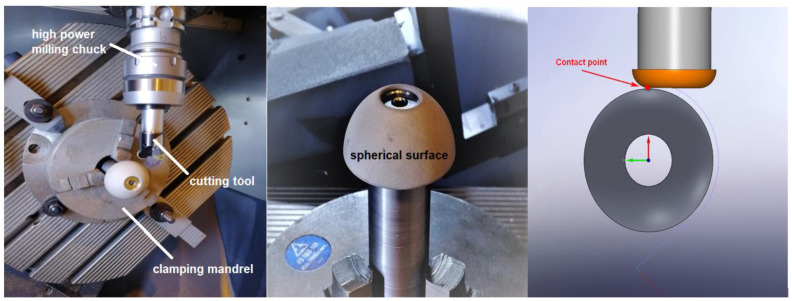
Linear milling centre DMU 40 eVolinear during milling of the examined sample. The sample is clamped by means of a mandrel in a universal chuck. The tool is clamped in a KFH (high power milling chuck).

In the presented experiment, a hemispherical steel surface with a diameter of 50 mm and a height of 15 mm (substrate) was covered with a coating based on NiCrBSi alloy. Figure 1 is a view of the concerned workpiece, including a 5-axis machining head.

Prior to spraying the coating on the substrate, thorough preparation is usually carried out, according to:

- the need for substrate treatment, i.e., degreasing, dirt removal, surface blasting;
- the method of surface treatment of the substrate, i.e., grinding, turning or milling;
- used technologies and required technological parameters of coating, i.e., the selected method and media of the coating process, material and additional material of coating, required thickness and roughness of coating; requirements for the structural arrangement of the coating with regard to its possible future applications, i.e., the required precision of surface treatment in terms of its dimensions and quality, as well as the method of clamping to achieve maximum uniformity of coating thickness after the coating process or other specific requirements.

### 2.1. Substrate—EN 10060 Steel ((34CrNiMo6)

Steel EN 10060 (34CrNiMo6) was chosen as the substrate, which is used for highly stressed machine parts with an emphasis on the required toughness, strength and hardness of the material. This material was purchased to make a ball and socket assembly. Table 1 shows the chemical composition of the substrate.

Table 2 lists the key parameters of a sample of the substrate used according to the tensile diagram (after a standardised tensile test), namely yield strength, contraction, ductility as quantified plastic deformability before reaching yield strength, yield strength of the material. Table 1 and Table 2 show the values from the material sheet. The material is considered a reference material and sufficient for the purpose of meeting the stated goal. The Material Certificate was provided by Schmolz + Bickenbach Ltd. (Kladno, Czech Republic).

Based on the above facts, in the presented case, the fact that the parameters of HVOF range at temperatures from 1000 to 3000 K and at velocities from 350 to 1100 m∙s^−1^ can be utilised; it suits both the presented experiment (substrate material with the melting point *T_melt_* = 3146 K), as well as the spray material (with the melting point *T_melt_ =* 1672 K).

### 2.2. Spraying Material—NiCrBSi Alloy

The coating was applied to the prepared substrate according to the established experimental requirements:

- chemical composition of the coating material;
- coating thickness: 0.5 mm;
- coating roughness: *Ra* = 7.5 μm;
- method used: High Velocity Oxygen Fuel (HVOF)—thermal spray coating process;
- coating media used: oxygen, kerosene;
- the carrier gas used to feed the powder consumable was argon,
- Ni-based alloy powder has a particle size of 20–53 µm, the hardness of HRC is 60. These data are measured by GTV Verschleißschutz GmbH, which performed a certified test according to EN 10204-3.1;
- machining of the generated surface by milling.

The chemical composition of NiCrBSi powder is given in Table 3. These data are measured by GTV Verschleißschutz GmbH (Luckenbach, Germany), which performed a certified test according to EN 10204-3.1.

**Figure 2:**
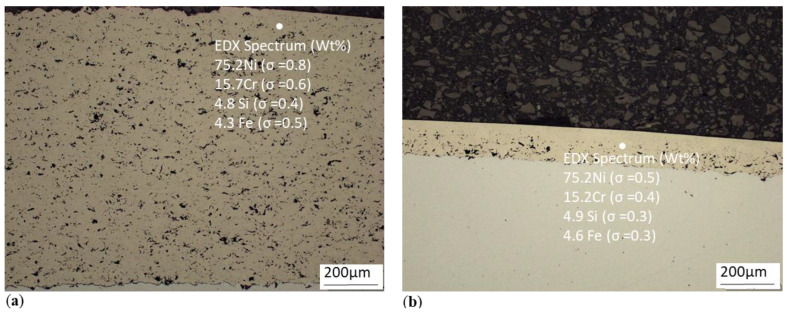
EDX analysis of NiCrBSi coating (**a**) before; (**b**) after machining.

Figure 2 shows the analyses of NiCrBSi powder injection before and after machining, obtained using the EDX (Energy-dispersive X-ray spectroscopy) method commonly used in electron microscopy (Figure 2a,b). Provided spectra are representative for each sample and were obtained at metallographic sections, 50 μm below the surface to eliminate the signal from the matrix. Light elements such as boron are hard to quantify by this method, and carbon contents are furthermore affected by common contamination of analysed surfaces. Therefore, those two elements were excluded from the spectra.

### 2.3. Basic Milling Parameters to Be Examined

In a set of experiments using NiCrBSi alloy spraying on a steel substrate, a total of 15 millings of the generated surface was performed on a DMU 40 eVolinear linear milling centre (DMG MORI, Pfronten, Bavaria, Germany) equipped with a headstock HSK-A-63 (max. 24,000 rev∙min^−1^, 25 kW). For the presentation of the machining of the selected sample, a combination of key technological parameters was chosen (Table 4): cutting speed *v_c_* (m∙min^−1^), feed per tooth *f_z_* (mm) and cutting depth *a_p_* (mm).

During the pilot test, two variants of milling were performed (consecutive and non-consecutive), in both cases a comparatively stable cutting process and a sufficient quality of the machined surface was achieved. Because non-consecutive machining reduces tool life due to increased cutting edge friction, further tests were performed only by sequential milling. The tool moved in a spiral with a constant step size over the machined spherical surface, thus ensuring even surface machining. At the beginning of each operation, the paths were generated on an auxiliary, tangentially connected surface, thus ensuring a smooth approach of the tool into the workpiece material. The path designed in this way ensured a constant removal of material along the entire path of the tool. This method is only suitable for machines that allow circular interpolation, i.e., machines that are numerically controlled. The side-step (feed size) was changed with a step of 0.05 mm, i.e., its values of 0.1, 0.15, 0.2, 0.25 and 0.3 mm were set successively. The depth of cut was limited by the thickness of the spray layer. The feed rate was defined as the feed per tooth and was changed in 0.05 mm∙tooth^−1^ increments, gradually setting its values to 0.1 to 0.3 mm∙tooth^−1^. Due to the fact that the material was removed in the tool axis, the feed rate was the same as the maximum chip thickness.

The values of the cutting parameters were chosen in combination, according to the positive empirical experience gained during the pilot milling of the spherical surface (Table 4). In experiments 1 to 4, the settings of the parameter vc were changed with constant settings of the parameters *f_z_*, *a_p_*. Furthermore, in experiments 5 to 15, the setting of a constant cutting speed was maintained, and the settings of the parameters feed per tooth and depth of cut were combined (except for the same setting of these parameters for the 7th and 15th experiments for checking purposes), so that for the given settings of the parameter fz, the settings of the parameter ap were changed in triplicate values of 0.20; 0.25; 0.3 mm (except for in experiment 6, in which the combination option was the only one (0.10 mm).

### 2.4. Fundamental Equations for Deformation Modelling

For the transverse roughness *Ra_q_* and the stress *σ_rzq_*, the following Equations (1) to (2) are used, (MPa):

(1) $$Ra_{q}\;=\;Ra_{0}\cdot {\left(\sqrt{\left(log{\left(h\right)}^{2}+log{\left({\left(h\cdot tg\delta \right)}^{1}\right)}^{0.25}\right)}\right)}^{0.333}\;$$

where *δ* is single of deviation from the vertical plane (MPa):

(2) $$\sigma_{rzq}=10^{-3}\cdot \;\frac{Ra_{q}\cdot E_{mat}}{Ra_{0}}$$

Both Equations are derived by the authors of the article, they are experimentally and statistically verified and have been published for many years, e.g., [28,29,30], etc., or are also the content of patents [31,32]. They are very important in our application solutions because they simulate laboratory tensile and compressive tests for diagrams *σ-ε*, or *σ-*Δ*h* with relatively precise localization of the position and the value of the yield strength *Re*, and thus also the position of the neutral deformation plane *h*_0_ and other stress-strain parameters of the materials.

The trace roughness *Ra* can be calculated in various ways, the simplest one being, e.g., according to Equation (3):

(3) $$Ra=\left(-10\right)\cdot \left(1-\frac{K_{plmat}}{K_{plmat}-h}\right)$$

for the neutral plane *h*_0_ (4):

(4) $$Ra_{0}=\left(-10\right)\cdot \left(1-\frac{K_{plmat}}{K_{plmat}-h_{0}}\right)$$

where in the deformation stress in the track is (5):

(5) $$\sigma_{rz}=Ra\cdot \frac{E_{mat}}{Rao}$$

What remains is to explain the method of exact calculation of the position of the yield strength *Re* and the neutral plane *h*_0_, which is valid in general for all technical materials, based on the decomposition of stress into tensile and compressive components:

modular tension component (6):

(6) $$E_{ret}=E_{mat}\cdot \frac{K_{plmat}}{\sqrt{Ra\cdot h}}$$

modular compressive component (7):

(7) $$E_{retz}=E_{mat}\cdot \sqrt{\frac{Ra\cdot h}{K_{plmat}}}$$

tensile stress component (8):

(8) $$\sigma_{ret}=\;\sqrt{E_{ret}}$$

compressive stress component (9):

(9) $$\sigma_{retz}=\sigma_{rz}=\;\sqrt{E_{retz}}$$

In the neutral stress-strain plane *h*_0_, similarly to the beam, the tensile (+) *σ_ret_* and compressive stresses (−) *σ_rz_* are equalised. Therefore, it is here at the *h*_0_ level that we find the minimums of the analysed functions/parameters in the analyses and graphs.

## 3. Results and Discussion

### 3.1. Analysis of Thermodynamics Functions

The destructive power of the laser in the studied material *W_las_* (kW) (10) at the laser-induced temperature *T_las_* (K) is applied as an energy source. Selected thermodynamic parameters are used for the presented solution, while the calculations are performed in connection with previously published literature [22,23,24]:

(10) $$W_{las}=\frac{824.933}{K_{plmat}^{2}}\cdot \left(622.392\;+408.832\cdot a_{p}-21.933\cdot a_{p}^{2}+\;0.485\cdot a_{p}^{3}\right)$$

where *W_las_* is laser power (W), the investigated parameters are altered deformability constants *K_plmat_* and the depth of cut *a_p_* (11):

(11) $$a_{p}=\frac{a_{p0}}{h_{0}}\cdot h_{cut}$$

where *h_cut_* is dept of cut (mm), *a_p_*_0_ depth of cut in the neutral plane (mm) and *h*_0_ is depth in the neutral plane

The parameters obtained from the laser are evaluated classically according to Equations (12) to (19), analogous to the thermal load developed during thermal spraying:

(12) $$T_{las}=\left(580+0.46\cdot W_{las}\right)$$

(13) $$T_{melt}=\sqrt{T_{las}\cdot \gamma_{m}}$$

where *T_melt_* is melting temperature of the material (K), *γ_m_* shear stress given by the temperature in the cut for the level *T_pm_* (N∙m^−1^);

(14) $$Q_{h}=W_{las}\cdot Ra\cdot t_{cut}$$

where *Q_h_* is heat according to the depth of cut *h_cut_* (J) and *t_cut_* is time of cutting (s);

(15) $$G_{h}=dH-T_{las}\cdot dS$$

where *G_h_* is Gibbs free energy according to the depth of cut *h_cut_*, or according to *a_p_* (J),

*dH* is enthalpy according to the depth of cut *h_cut_*, or according to *a_p_* (J),

*dS* is entropy according to the depth of cut *h_cut_*, or according to *a_p_* (J∙K^−1^).

(16) $$dH=dS\cdot T_{las}$$

(17) $$dS=\frac{W_{las}\cdot t_{cut}}{T_{las}}$$

(18) $$Q_{h}=W_{las}\cdot Ra\cdot t_{cut}$$

where *Q_h_* is heat according to the depth of cut *h_cut_*, or according to *a_p_* (J),

and heat flux according to the classical Equation (19):

(19) $$q=\frac{Q_{h}}{dS\cdot t_{cut}}$$

Therefore, parameters *Hdt*, *Udt*, *Qdt*, *Gdt*, *Sdt* are calculated here as increments at time *t* = *t_cut_* (s). In the text, after the calculation according to Equations (10) to (19), these parameters are, subsequently, regressively converted into relations to one independent variable, which is the deformation increment at time *t* (s) and denoted as *Hdt*, *Udt*, *Qdt*, *Gdt*, *Sdt*. This is because the deformation time can be easily converted to the deformation rate v (m∙s^−1^). Or possibly, also to the deformation length *h*, *h_cut_* (mm), to the relative deformation length *h_rel_* (−) or to the depth of engagement of the machining tool *a_p_* (mm). The parameters *Hdt*, *Udt*, *Qdt*, *Gdt*, *Sdt* are, therefore, calculated here as increments at time *t* = *t_cut_* (s). Based on the results from physical Equations (10) to (19), they are regressively transformed into polynomial according to the time *t* of the laser passing through the material. In this way, continuous distributions to other parameters can be obtained because *t_cut_* = function (*a_p_*, *h_cut_*, *v_cut_*, *T_las_*, *R_mp_*,…). The computational polynomials for the given increments at time *t*, *t_cut_* are expressed in regression Equations (20)–(25):

(20) $$t=t_{cut}=\;\frac{h_{cut}}{13.94\cdot \sqrt{\frac{35.77}{K_{plmat}}}}$$

(21) $$Hdt=\;691.245\;+\;9574.144\cdot t\;-176.724\cdot t^{2}-17326.476\cdot t^{3}+\;11898.485\cdot t^{4}$$

(22) $$Udt=594.272\;+\;11845.853\cdot t\;-14138.717\cdot t^{2}+\;6650.286\cdot t^{3}-456.513\cdot t^{4}$$

(23) $$Qdt=594.272\;+\;11845.853\cdot t\;-14138.717\cdot t^{2}+\;6650.286\cdot t^{3}-456.513\cdot t^{4}$$

(24) $$Gdt=-359.584\;+\;1276.375\cdot t\;-1815.213\cdot t^{2}+\;1229.102\cdot t^{3}-357.681\cdot t^{4}$$

(25) $$Sdt\;=\;0.271\;-1.442\cdot t\;+\;2.696\cdot t^{2}-2.115\cdot t^{3}+0.601\cdot t^{4}$$

If the value *t*_0_, which is valid for the neutral plane *h*_0_, is substituted for the variable *t* into the Equations (21)–(25), it is easy to calculate the given parameters in the forms of potentials *Hdt*_0_, *Udt*_0_, *Qdt*_0_, *Gdt*_0_, *Sdt*_0_, which can then be determined as continuous potentials according to specific cutting parameters, using the function *R_mp_* = function (*v_c_*, *f_z_*, *a_p_*, *v_c_*_0_, *f_z_*_0_, *a_p_*_0_).

### 3.2. Processing the Results of Calculations According to the Assignment of the Experiment

Due to the mathematical application of the *R_mp_* parameter, it is possible to solve all important functions that are related to the topics addressed in the presented work accurately and very easily. Calculations based on the *R_mp_* parameter are performed according to the original relations, which were derived due to the need for a simple method for the exact mathematical expression of the bond in a multi-parametric system.

In particular, the dimensionless-relative parameter *R_mp_* (26) was newly introduced as a parameter with a comparative information value, i.e., as the ratio of the machining parameters selected and the machining parameters on the neutral plane *h*_0_ (26):

(26) $$R_{mp}=\frac{R_{mpX}}{R_{mp0}}$$

where *R_mpX_* is the selected set of main cutting parameters (27):

(27) $$R_{mpX}=f_{z}\cdot v_{c}\cdot a_{p}$$

and *R_mpo_* is a set of main cutting parameters for the neutral plane (28):

(28) $$R_{mp0}\;=\;f_{z0}\cdot v_{c0}\cdot a_{p0}$$

where *f_z_*_0_ is the feed per tooth on the neutral plane (mm).

**Figure 3:**
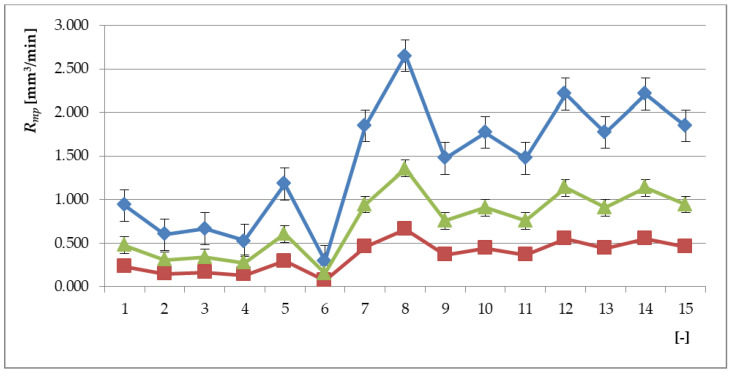
Graph of *R_mp_* = function (*E_xpNO_*) values calculated according to Table 3 for *R_mpSUB_* substrate materials (blue), *R_mpXY_* interlayers (green), and *R_mpX_* overlay spraying layers (red); it can be observed that at the level of *E_xpNO_* = 6, the curves of all three materials converge to the value *R_mp_* > 0.

Values of *R_mp_*, *R_mp_*_0_ and *R_mpX_* for spraying material NiCrBSi as the function (*E_xpN_*_0_) as specified in Table 3 are declared in Figure 3. The parameter *R_mp_* (26) can be used relatively easily to calculate all functions related to machining. By making the parameters *f_z_*, *v_c_*, *a_p_* independent, it is possible to precisely determine the value of *R_mpX_* for a set of selected cutting parameters by retrospective calculation or to determine individual parameters and their influence on the result separately and partially. For dimensionless-relative parameter containing surface roughness *R_mpRa_*, the following Equation applies (29):

(29) $$R_{mpRa}=\left(\frac{f_{z}}{f_{z0}}\cdot \frac{v_{c}}{v_{c0}}\cdot \frac{a_{p}}{a_{p0}}\cdot \frac{Ra}{Ra_{0}}\right)=\frac{R_{mp}}{R_{mp0}}\cdot \frac{Ra}{Ra_{0}}$$

where *Ra*_0_ is the surface roughness (µm) on the neutral plane.

From ratio formula (29), for the calculation of surface roughness, the following Equation applies (30):

(30) $$Ra=Ra_{0}\cdot \frac{R_{mpRa}}{\left(\frac{f_{z}}{f_{z0}}\cdot \frac{v_{c0}}{v_{c0}}\cdot \frac{a_{p0}}{a_{p0}}\right)}=Ra_{0}\cdot \frac{R_{mpRa}}{R_{mp}}$$

For dimensionless-relative parameter containing surface adhesiveness R*_mpAdh_*, the following applies (31):

(31) $$R_{mpA_{dh}}=\left(\frac{f_{z}}{f_{z0}}\cdot \frac{v_{c}}{v_{c0}}\cdot \frac{a_{p}}{a_{p0}}\cdot \frac{A_{dh}}{A_{dh0}}\right)==\frac{R_{mp}}{R_{mp0}}\cdot \frac{A_{dh}}{A_{dh0}}$$

where *A_dh_*_0_ is the adhesion on the neutral plane (MPa).

From ratio formula (31), for the calculation of surface adhesion, the following applies (32):

(32) $$A_{dh}=A_{dh_{0}}\cdot \frac{R_{mpA_{dh}}}{\left(\frac{f_{z}}{f_{z0}}\cdot \frac{v_{c}}{v_{c0}}\cdot \frac{a_{p}}{a_{p0}}\right)}=A_{dh_{0}}\cdot \frac{R_{mpA_{dh}}}{R_{mp}}$$

Table 5 shows the results of the calculated *R_mp_*. Here, the minimums of, e.g., the parameter *R_mp_*, are altered constants of deformability *K_plmat_*, roughness *Ra*, entropy *dSdt*, adhesion *A_dh_*, diffusion *D_iff_*.

Figure 3 plots the distribution characteristic and diffusion intensity discretely as specified in Table 3.

**Figure 4:**
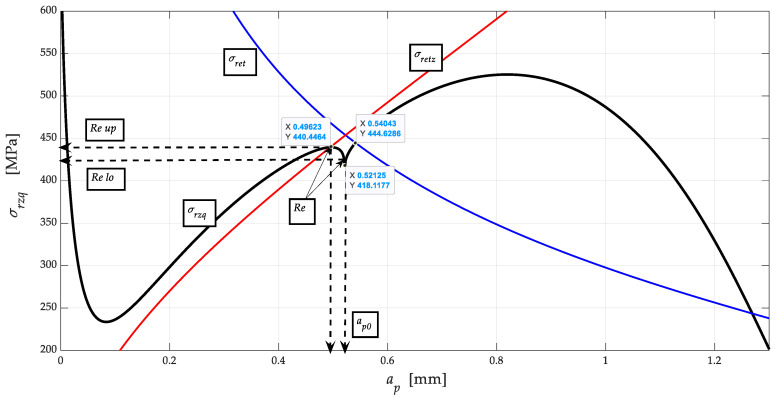
Dependence of stress *σ_rzq_* on the depth of cut *a_p_.*

Figure 4 shows in detail the shapes and sizes of the upper and lower *R_eX_* limits for the yield strength of the NiCrBSi spraying layer, as well as a method for exact determination of the position of the yield strength *R_eX_* and the position of the neutral plane *h*_0*X*_ on the main stress-strain envelope *σ_rzqX_*-*a_pX_*. The main stress-strain envelope *σ_rzqX_*-*a_pX_* is generally most often calculated as a function of *Ra_q_* roughness, whose equation is morphologically more complex but precisely outlines the shape, position, and size of *Re* = function (*Ra_q_*). Therefore, we distinguish two types of roughness. Roughness in the track of the machining tool *Ra*, where the position, shape, and size of the upper and lower yield strength are not reflected in the measurement or calculation only becomes apparent when calculating and measuring the distribution of *Ra_q_* roughness across tool marks. It should be noted here that the symbol for the depth of cut *a_p_* (mm) is used in chip machining technologies and thus with a fixed tool/blade. For machining/cutting using AWJ, laser, plasma, etc. technologies, the symbol for the deformation length *h* (mm) is used for the depth of cut, where the achievable limit depth of cut is *h_lim_* = *K_plmat_* (mm). The conversion is given by the dimensionless ratio *K_hap_* = *a_p_*_0_/*h*_0_ (−).

Figure 4 shows the dependence of the stress *σ_rzq_* on the depth of cut *a_p_* in accordance with Equations (1) and (2). These Equations were used to identify the stress-strain state of the thermally stressed material. The curve (black) shows local extremes that can be semantically assigned two entropy minima and two enthalpy maxima. It is in these localities that the interconnection through Young’s modulus of elasticity is clearly evident. The relationship between stress, entropy and enthalpy in a significantly thermally stressed material is evident through Equation (15). This material modulus is significantly dependent on the temperature developed during significant stress deformation (2). The position of the yield strength *Re* and the neutral plane *h*_0_ is marked by the intersection of the functions *E_ret_* and *E_retz_*. Likewise, *Re* and *h*_0_ are given by the intersection of the stress functions *σ_ret_*, or *σ_retz_* = *σ_rz_*.

**Figure 5:**
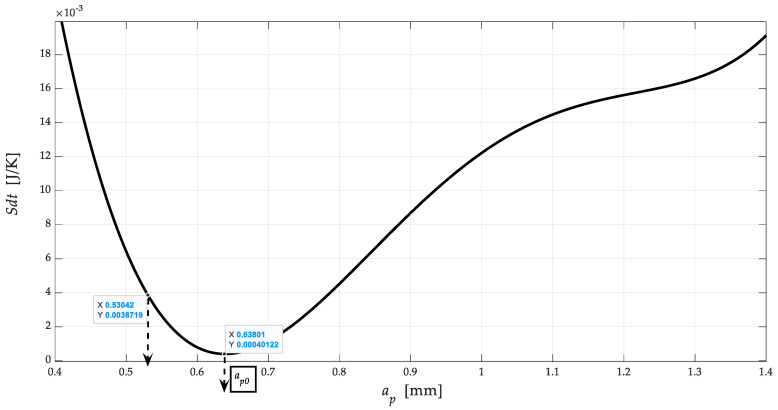
Detail of the course of entropy distribution *Sdt*-*a_p_*. Depth of engagement *ap*, or neutral engagement *apo* under a fixed tool are in the function of deformation lengths (*a_p_*, *a_p_*_0_) = fce(*h*, *h*_0_).

Figure 5 shows a detail of the course of the entropy distribution *Sdt*-*a_p_*. In the presented cases of the dependence of entropy on the depth of cut, the occurrence of two local minima is obvious. These courses can be generalized, and it can be predicted that the course of the curve will be analogous for other entropy dependences on the depth of cut. The first local minimum is on the neutral plane, in which the compressive stress and the tensile stress are balanced, and thus the entropy is minimal due to the relatively best order of the system. The second local minimum corresponds to the yield point, where, in terms of mechanical properties, there is an accumulation of energy needed to change the shape. On the contrary, the Gibbs curves of the free energy distribution *Gdt*-*a_p_* reveal minima at the level of the plane for the strength limit *Rm*, which can also be logically justified from a mechanical point of view.

### 3.3. Layering of the Coating on the Substrate

In principle, after thermal spraying (with a flame thickness of 1–3 mm), the coating is remelted, and a diffusion bond is formed, i.e., a metallurgical bond of the coating with the substrate, this coating becoming highly entropic. In solving the problem of layering the coating on the substrate, we further confirmed that analytically, there are at least three different layers of material. It is a base layer of substrate that is subjected to melting. The melting of the substrate is accompanied by the formation of lower and upper ionic transition layers. Only on this layer, in the final phase of cooling and solidification, the upper layer given by the powder coating material is formed. After HVOF application, the substrate is additionally heated; the whole part is heated to a temperature of 1000 to 1100 °C.

Based on the presented original analytical procedures, it is possible to differentiate the individual layers well, especially in terms of physical-mechanical and thermodynamic parameters of these layers. The described layering is thus well observable on the finished experimental workpiece due to the colour difference of the layers (e.g., under a magnifying glass or better under a microscope). The distribution, according to the layer thickness in mm, can be measured easily, and the presented calculations can be verified independently.

### 3.4. Entropy Theory

**Figure 6:**
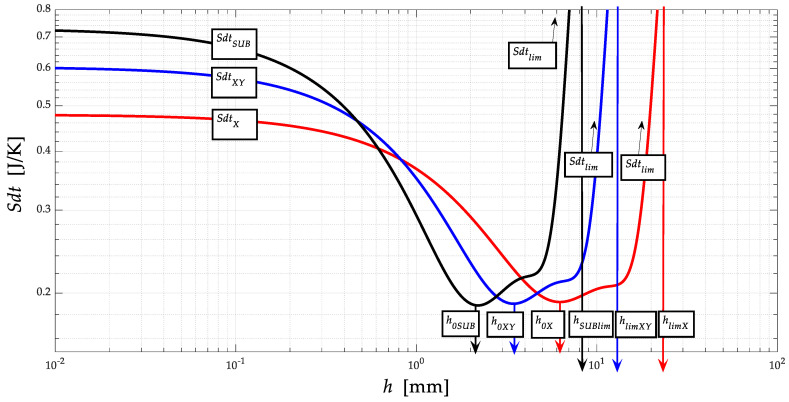
Distribution curves for substrate, for XY-layer transition material, and for spraying material (*Sdt*) = (*h*).

Within the presented solution, the distribution functions *Sdt* = function(*h*), or *Sdt* = function(*a_p_*) (J∙K^−1^) are calculated, so very interesting connections between physical-mechanical deformation parameters and thermodynamic parameters become apparent. Figure 6 plots the distribution curves for the substrate, for the XY-layer transition material, and for the spraying material. It can be seen that in all cases, the *dSdt* curves converge to zero, exactly at the level of the yield strength *Re* of the neutral planes *h*_0_ for the given materials. Furthermore, it is derived that at the level of neutral planes *h*_0_, the value of entropy *Sdt* is minimal, because, at *h*_0_, the tensile and compressive deformation stresses equalise during the deformation of the material, so the equality that (+) *σ_ret_* = (−) *σ_retz_* applies.

Details of the distribution of entropy curves *Sdt* (Figure 6) at the level of neutral deformation lengths ho for substrate *h*_0*SUB*_ = 2.24 mm, for XY-layer transition material *h*_0*XY*_ = 3.51 mm, and for spraying material *h_oX_* = 6.37 mm are plotted. This illustrates our new finding that at the *h*_0_ level for all materials, the entropy *Sdt* converges to zero. We deduce that it is at the level of the neutral plane *h*_0_, where the stress components (+/−) stress/pressure are equal, that the resulting stress in the deformed material is zero. The same is true, for example, in the beam theory. Both the stress-strain and thermodynamic processes inside the materials are stopped.

**Figure 7:**
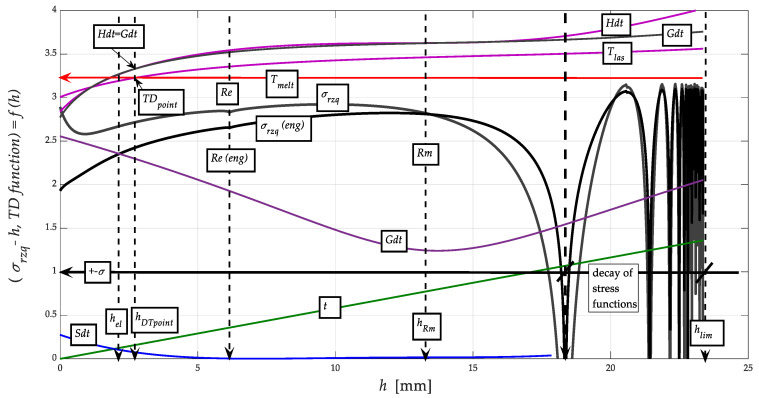
Distribution curves of stress-strain and thermodynamic functions (entropy *Sdt*, Gibs *Gdt*, energy *Hdt*, *Qdt*) at depth *h*.

**Figure 8:**
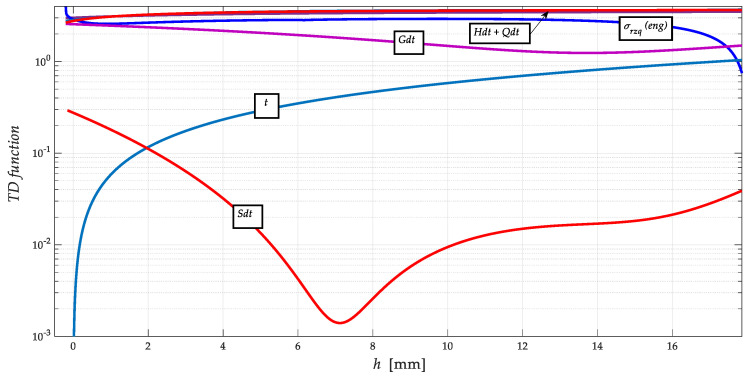
A detail of distribution curves of thermodynamic functions (entropy *Sdt*, Gibs *Gdt*, energy *Hdt*, *Qdt*) at depth *h*.

The results of the calculation of selected parameters according to Equations (10) to (20) and their graphical processing are shown in Figure 7 and Figure 8. Only the functions related to NiCrBSi spraying material are plotted; they are similar for the substrate and the intermediate layer.

It can be clearly seen in Figure 8 that the Gibbs free energy distribution curve *dGh*, on the other hand, has a minimum at the level of the ultimate tensile strength *R_m_* of the stress-strain curve *σ_rzq_* = function(*h*). The rationale for this phenomenon can be seen in the fact that at the level of reaching *R_m_*, the maximum free energy is depleted to maintain the homogeneity/stability of the structure of the stressed material. Figure 8 shows a detail of the distribution curve of the *dSh* entropy function. Here it can be seen very well that its minimum converging to zero is exactly at the level of the yield strength *Re*, and thus in the neutral plane of the deformed material, or in the presented case of the spraying material, i.e., where both stress equilibrium (+)*σ_ret_* = (−)*σ_retz_* and modulus equilibrium (+)*E_ret_* = (−)*E_retz_* apply. Both of these findings may be important for further study of thermodynamic and material parameters/properties of materials.

### 3.5. Construction Graph of the Thermo-Mechanical State after Thermal Spraying

Substrate EN 10060 (34CrNiMo6) steel and spraying material NiCrBSi are, according to their chemical composition, multi-element materials, which, when combined with other materials, especially in the molten state, form desirable highly entropic mixtures during thermal spraying. The high level of entropy induces the desired intensity of ion redistribution in the recrystallisation process, as evidenced by the measurements according to Figure 2 in Section 2.2.

**Figure 9:**
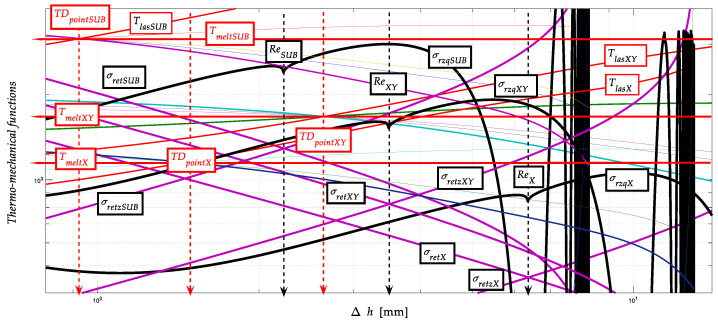
Modelled thermo-mechanical curves (thermal point *TD_point_*, melt point *T_melt_*, stress *σ_ret_*, yield point *Re*) after thermal spraying at the dependence on depth *h*.

The intensity of ion redistribution follows the entropy distribution according to Figure 5, Figure 6, Figure 7 and Figure 8. It is, therefore, possible to write implicitly energy in time *W_t_* = function (d*T*, d*S*, d*t*…). The original construction of the thermomechanical state after thermal spraying is shown on the basis of the above justifications, namely in Figure 9.

**Figure 10:**
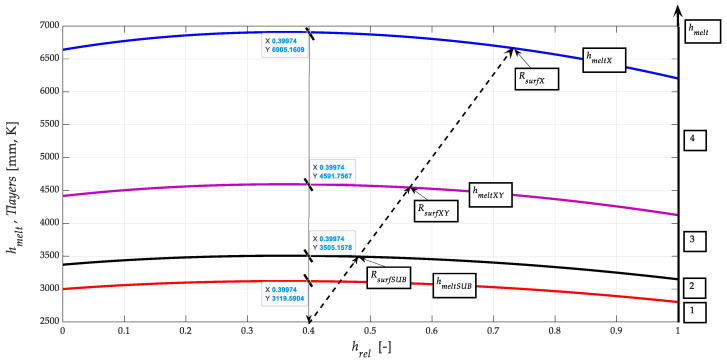
Distribution of the interface depth layers *h_melt_* classified by *T_melt_* at the relative depth *h_rel_.*

We are introducing distribution curves so that we can observe multiple dependencies at the same time in order to make the concerned dependencies readable within a single graph. We claim that the results of the calculation of selected parameters according to Equations (1)–(11) and their graphical representation are shown in Figure 8 and Figure 9, just as stress-strain distribution curves and thermodynamic functions at depth *h*, Figure 10 relates to the construction of thermomechanical condition after heat spraying. This description, which is understandable, concerns only the distribution of free energy and the entropic function.

Figure 10 is relatively complex; however, due to the effort to capture the interrelationships of functions, it was used to show the triple layering between the materials of a very strong substrate and a softer coating, which, however, acquires hardness in the phases of recrystallisation solidification due to ion redistribution. In the middle between them, an intermediate layer of transition material is drawn. However, the mechanical parameters of all three material layers can be known to us because they can be exactly identified on the basis of current knowledge. The values of the melting points *T_meltE_*, the position of the thermodynamic intersections *TD_points_* and the values of the yield strength *Re* are very suitable and relatively easy to use for identification. Subsequently, the tensile modulus of elasticity *E_mat_* can be derived for individual layers of a given three-component material bonding system.

The inspiration is offered by the diagram in Figure 10, where the material interlayers are further divided into under-layer and top-layer with their own transitions. Of interest is the upper part of the intermediate layer referred to as the top-layer and the adhesive-layer adhering to it. It is well indicated here that the adhesive strength and thus the stiffness of the adhesive bond is given by the suitable roughness *Ra*, or *Rz* of the surface of the substrate after its machining, and thus a suitable ratio between the size of the structural grain *D_gr_* of the coating spraying material. A suitable *Ra*/*D_gr_* ratio [29] limits the porosity and any decrease related to homogeneity and stiffness of the transition, which fully corresponds to the original predicted theoretical consideration expressed by the Equation (33):

(33) $$A_{dh}=\;10^{-2}\cdot \frac{Ra}{D_{gr}}\cdot \sqrt{E_{mSUB}\cdot E_{mCOV}}\cdot kpor$$

The values of the parameters in the Equation (32) can be determined relatively simply and discretely:

*A_dh_*  Adhesive stress, i.e., the adhesive force acting on the grain area of the coating (MPa),
*D_gr_*  Size/diameter of the structural grain of the coating (µm),
*E_mSUB_*  Young’s modulus of elasticity of the substrate material (MPa),
*E_mCOV_*  Young’s modulus of elasticity of the coating/cover material (MPa),
*k_por_*  degree of porosity of the coating according to the spraying technique (–).

In conclusion, it should be noted that the transition material layers, including recrystallisation processes in the field of thermal spraying, are often groundlessly neglected in the literature and in practice.

### 3.6. Results Verification

Technologically correct and optimally selected composition of the main cutting parameters immediately creates the removed volume of the machined material, the structure and texture of the machined surface, the instantaneous stress-strain and thermodynamic state at the contact between the tool and the surface layer of the material, in the case of thermal spraying, also the specific state of recrystallisation quantities at the contact of the substrate and the spraying. The mutual layering of the spray layers, thermodynamic and mechanical transitions, including the thermo-mechanical state of the transition layers, were investigated in more detail and analytically.

The theoretical way of solving mechanical and thermodynamic problems of the spraying process can be well verified on such a workpiece because, at the edges of the workpiece, differentiated interfaces of individual layers are clearly visible under a magnifying glass or microscope; therefore, layer thicknesses are well measurable. The thicknesses of the layers marked (1 to 4) can be used to verify the theoretically calculated material and dimensional parameters of the individual coating layers. The verified values of the thicknesses of the individual layers are distributed in Figure 10.

The calculated identification data of the selected layer parameters are given in Table 6.

Table 6 also includes the diffusion parameter in the neutral plane *D_iff_*_0_ (kg∙m^−2^∙s^−1^). The value *D_iff_*_0_, or *D_iff_* continuously as the function of cutting parameters, i.e., as the function *R_mp_*, or diffusion as a phenomenon of spontaneous dispersion of particles cannot be omitted in the investigated case of fusible alterations of substrate materials and interlayer spraying. The value of the parameter *D_iff_*_0_ in the plane *h*_0_ can also be referred to as the diffusion potential of the material. The diffusion parameters in the contact melting process are adjusted according to Fick’s theory so that it is possible to use the parameter *R_mp_* as the function to the cutting parameters *R_mp_* = function (*f_z_*, *v_c_*, *a_p_*/(*f_zo_*, *v_co_*, *a_po_*) (34) to (35):

(34) $$R_{mpdif}=\left(\frac{f}{f_{o}}\cdot \frac{v_{c}}{v_{co}}\cdot \frac{a_{p}}{a_{po}}\cdot \frac{D_{iff}}{D_{iffo}}\right)\;\;$$

(35) $$D_{iff}=\frac{D_{iffo}\cdot R_{mpdif}}{\frac{f}{f_{o}}\cdot \frac{v_{c}}{v_{co}}\cdot \frac{a_{p}}{a_{po}}}$$

where *D_iffo_* is the function of the laser temperature *T_laso_*, or *W_laso_* laser power in the *h_o_* plane (36):

(36) $$D_{iffo}=\omega_{o}\cdot k\cdot T_{las}$$

where *ω*_0_ parameter is given by the Equation (37):

(37) $$\omega_{0}=\;\frac{v_{cut0}}{F_{cut0}}$$

and also, according to Fick’s theory, the parameter for the force is defined as *F_cut_*, which is given by the Equation (38):

(38) $$F_{cut}=\frac{\rho \cdot g\cdot h}{h_{o}^{3}}$$

where *ρ* is the density and *g* s the gravitational acceleration.

Finally, the melting depth *h_melt_* was derived using the *T_meltE_* function related to the material parameter *E_mat_* (39):

(39) $$T_{meltE}=\frac{E_{mat}-46\;365.359}{95.590}$$

so the Equation (40) applies:

(40) $$h_{melt}=0.823\cdot \left(0.0000002\cdot T_{meltE}^{2}-\;0.001\cdot T_{meltE}\;+\;2.712\right)$$

**Figure 11:**
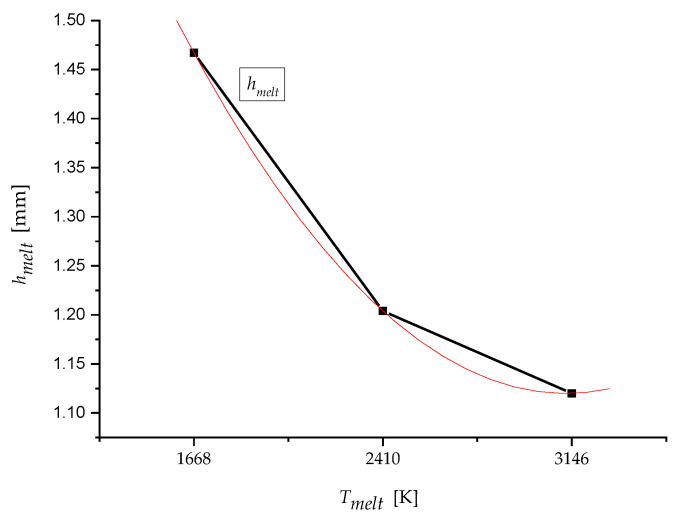
The dependence of the melting depth *h_melt_* on the melting temperature *T_melt_*.

The derived parameter melt depth *h_melt_* (mm) will be very much needed in many other applications of technical practice. The graph for the melting depth *h_melt_* is a polynomial of the 2nd degree, according to Figure 11, where the total accuracy *R*^2^ = 1 (39).

In the case of the presented experimental hemispherical sample, the total melting depth is summarised for the represented contact materials at 3.792 mm. This value can be verified by direct measurement (under a magnifying glass or microscope) directly on the finished experimental product. On this basis, a verification graph was then processed (Figure 10).

**Figure 12:**
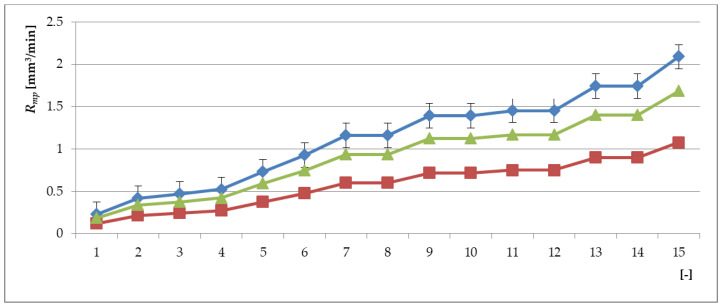
Modelled curved of *R_mp_* (*R_mpSUB_* substrate materials—blue, R*_mpXY_* interlayers—green and *R_mpX_* overlay spraying layers—red) depending on the number of experiment *E_xpNo_*.

Figure 12 shows the distribution characteristics according to Equation (34) for the individual examined parts: the substrate, the intermediate layer and the final surface. The interaction of the interlayer particles and the final surface is declared in Figure 9 (mechanical and thermodynamic properties related) and 10 (distribution of layer levels).

### 3.7. New Results and Discussion

Selected analytical procedures to achieve the target outputs of the presented solution are described above. In short, these are analytical methods for identifying the distribution functions of entropy *dS*, neutral planes *h*_0_, including the method of detailed identification of upper and lower yield strength *Re*, material layering, and thermodynamic points *TD_points_* of superimposed layers of thermal spraying, adhesion potentials *A_dh_*, the thickness of molten coating layers *h_melt_*, and finally, their final strength parameters, which are essential for application in technical practice. The set of these newly analysed parameters of interest is specified in Table 5. At the same time, connections between the identified thermodynamic and mechanical functions were sought. Based on exact data and graphs, mostly according to the authors’ newly derived equations, a number of new findings were obtained, which can be briefly formulated into the following points:

(a) physical-mathematical formulation of layering of molten materials at the contacts of the substrate, interlayer, spraying;
(b) physical-mathematical formulation of the process and melting mechanism in the thermal spraying technology;
(c) physical-mathematical formulation of alteration of materials on contact in terms of mechanics and thermodynamics;
(d) alteration of materials on contact in terms of mechanical and ionic redistribution of the structure;
(e) derivation of a number of new multi-parametric equations with general validity and significant application potential;
(f) verification and confirmation of the significance of the new dimensionless parameter Rmp for application calculations.

## 4. Conclusions

The aim of the presented article was to predict and classify the technological parameters of milling a spherical substrate made of EN 10060 steel after spraying HVOF with NiCrBSi nickel alloy. Based on the analytical processing and interpretation of measured data from a set of 15 milling experiments, a broader database of results in tabular and graphical form was generated and verified, and the conclusions reached were interpreted. The set of selected technological parameters of milling was selected for the given experiment according to the assignment (Table 3).

The selected cutting parameters can be considered technologically fundamental because they directly determine the volume of material removed by milling, as well as in other chip-forming machining technologies. They determine the structure and texture of the machined surface, as well as the state of the instantaneous adhesive stress between the substrate and the coating, the diffusion parameters of melt vapour, mechanical and thermodynamic alteration of material contact layers, and the instantaneous stress-strain state of the affected volume at tool-material contact.

The newly derived distribution functions of selected cutting parameters (*R_mp_*, *R_mp_*_0_) = function (*v_c_*, *v_c_*_0_, *f_z_*, *f_z_*_0_, *a_p_*, *a_p_*_0_) are, in principle, absolute chip removals. They are of great importance for the calculation of relative technological and thermodynamic parameters of machining and thermal spraying. They enable calculations and graphical constructions of distribution functions in connection with the depth of cut *a_p_* and, in case of machining, or with the depth of cut *h* in case of continuous cuts by AWJ, laser, plasma, etc., using current abrasive technologies. It is a complex of physical-mechanical and new thermodynamic functions describing the mechanism of the machining process in connection with the melting processes given by the thermal spraying of the substrate. The presented original results relate mainly to the parameters listed in Table 5. Through the predicted and verified results, it was also clearly confirmed how to thoroughly address the effect of temperature changes on the change of physical-mechanical parameters, and predict the settings of technological parameters in connection with thermodynamic parameters, both for sample preparation and for the subsequent production of high-quality coatings.

## Figures and Tables

**Table 1:** Chemical composition of Steel EN 10060 [21].

Chemical Elements	C	Si	Mn	P	Cr	Mo	Ni	Cu	Sn
wt %	0.34	0.36	0.58	0.01	1.62	0.29	1.63	0.06	0.005

**Table 2:** Tensile tests performed at 296 K [21].

Mechanical Parameters	*Rm* ^1^	*Rp*_0.2_^1^	*A*_5_^1^	*Z* ^1^
	MPa	MPa	GPa	%
EN 10060	1024	932	17.3	62

^1^*Rm*—material strength, *Rp*_0*.2*_—yield strength, *A_5_*—ductility, *Z*—contraction.

**Table 3:** Chemical composition of NiCrBSi powder.

Chemical Elements	B	Co	Cr	Fe	Ni	O_2_	P	Si	Others
wt %	3.25	0.05	15.19	3.80	Bal	0.03	0.09	4.55	0.34

**Table 4:** Basic cutting parameters: cutting speed *v_c_*, feed per tooth *f_z_*, the depth of cut *a_p_* [30].

*E_xpNo_*	*v_c_* m∙min^−1^	*f_z_* mm	*a_p_* mm
1.	700	0.15	0.15
2.	450	0.15	0.15
3.	500	0.15	0.15
4.	400	0.15	0.15
5.	500	0.20	0.20
6.	500	0.10	0.10
7.	500	0.25	0.25
8.	500	0.30	0.30
9.	500	0.20	0.25
10.	500	0.20	0.30
11.	500	0.25	0.20
12.	500	0.25	0.30
13.	500	0.30	0.20
14.	500	0.30	0.25
15.	500	0.25	0.25

**Table 5:** *R_mp_* parameter for the *R_mpSUB_*, substrate, the *R_mpXY_* interlayer, and the overlay spraying layer *R_mpX_*.

*E_xpNo_*	*R_mpSUB_* mm^3^∙min^−1^	*R_mpX_* mm^3^∙min^−1^	*R_mpXY_* mm^3^∙min^−1^
1.	0.932	0.232	0.477
2.	0.596	0.1548	0.305
3.	0.666	0.166	0.341
4.	0.531	0.132	0.272
5.	1.179	0.294	0.604
6.	0.295	0.073	0.151
7.	1.846	0.460	0.946
8.	2.653	0.661	0.360
9.	1.474	0.367	0.756
10.	1.769	0.441	0.907
11.	1.474	0.367	0.756
12.	2.211	0.551	1.133
13.	1.769	0.441	0.907
14.	2.211	0.551	1.133
15.	1.846	0.460	0.946
Median	1.430	0.360	0.730

**Table 6:** Layers identification of mechanical and thermomechanical parameters.

Parameters	*E_mat_*	*Re*	*T_melt_*	*dSdt*_0_	*dG_min_*	*D_iff_*_0_	*a_p_*_0_	*HV*_0_	*A_dh_*_0_	*dQdt*	*h_melt_*
Layer	(MPa)	(MPa)	(K)	(J∙K^−1^)	(J)	(kg·m^−2^·s^−1^)	(mm)	(MPa)	(MPa)	(J)	(mm)
LayerX	206,000	417	1668	0.191	1.241	0.163	0.53	159	467	3350	1.467
LayerXY	276,740	651	2410	0.189	1.241	0.35	0.35	190	844	2064	1.204
Base	347,050	914	3146	0.188	1.241	0.416	0.22	229	1007	1397	1.12
